# Supplementary material for: Recommendations for Transdisciplinary Professional Competencies and Ethics for Animal-Assisted Therapies and Interventions
Source: Vet Sci. 2021 Dec 2;8(12):303. doi: 10.3390/vetsci8120303 (PMC8706351; doi:10.3390/vetsci8120303)
Supplement: Supplementary file 1 [file vetsci-08-00303-s001.zip › vetsci-1425476-supplementary.pdf]

Supplementary Materials

Summary of Considerations for APA Ethical Standards Competencies in Animal-Assisted Interventions Submitted by the Human-Animal Interactions Ethics Workgroup

Members: Amy Johnson, EdD, MA, MAT, LPC, CPDT-KA, UW-AAB
Rise VanFleet, PhD, LP, RPT-S, CDBC
Leslie Stewart, PhD, LPC
Susan Crowley
Molly DePrekel MA, LP
Emily Eccles, PhD, M.A.
Laura Hey, BAS, Certified Animal Assisted Intervention Specialist
Ann R Howie, MSW
Cynthia Johnson, Ph.D.
Betz King, Psy.D., L.P.
Natalie Runge
Sonya Snyder, MPhil, MSSW
Melissa Trevathan-Minnis, Ph.D.

ABOUT THE DIVISION

Section 13, Division 17 of the American Psychological Association, is an organization whose members conduct scholarly and professional activities that advance the field of human-animal interactions in psychology. This Section features an open-access, online peer-reviewed publication entitled the Human-Animal Interaction Bulletin (HAIB) and offers free continuing education webinars for Section members.

PURPOSE FOR THESE GUIDELINES

Without a set of guidelines, the public and professionals will make up their own or practice without guidelines. By providing a set of competencies within the field of psychology, practitioners can make informed decisions about their ability to participate in work associated with animal-assisted interventions (AAI), while maintaining a safe and effective practice. The overall goal for Competencies related to Animal-Assisted Interventions is to provide a framework for practitioners to ensure evidence-based practices and optimal welfare, particularly for animal partners.

This document is a ***guide*** for individuals and organizations who wish to practice in this field with a therapy animal (see Supplementary Materials for animal designations). **Competencies in this document are not meant to be used or presented as a credentialing tool**. At this time, there are no industry-wide standards of best practice. Competencies speak to a necessary fund of knowledge, while credentialing is specific to credentialing organizations. No one program will produce a clinician who satisfies all of the competencies. This includes degree and non-degree professional and continuing education, training, and supervision. The practitioner also recognizes that AAIs are an enhancement to the treatment process rather than as a stand-alone intervention.

This document applies the Animal-Assisted Interactions component of practice, after the assumption that competency as a psychologist has already been met. Standard 2.01 in the APA Code of Ethics asserts that professionals operate only within their scope of practice and boundary of competence related to populations, cultural sensitivity, new techniques/areas. Where standards do not yet exist, places the onus on the psychologists to take 'reasonable steps' to ensure competence, especially in animal behavior and species-specific and individual-specific communication (Chandler, 2018) .

Being proficient in one area does not automatically constitute proficiency in another. Adding a sentient being into the therapeutic milieu requires a specialized skill set that includes the knowledge, skills, and attitudes expected to continuously provide and improve the safety and efficacy within the field of AAIs (Stewart et al., 2014; VanFleet & Faa-Thompson, 2017). Although partners in AAIs, humans and animals do not share privilege or power, and therefore animals should be viewed as the more vulnerable species. As a result, therapy animal welfare requires the highest priority. This level of attention to ani-

mals ensures the safety and well-being of the client, animal, and practitioner. Furthermore, practitioners should understand the implications of social and cultural factors in clients' participation in AAIs.

This set of competencies stems from an empirically based study of AAI professionals conducted by Dr. Leslie Stewart using a Grounded Theory model. These standards have set the framework for competencies adapted and endorsed by Pet Partners and Animal Assisted Interventions International as well as the inclusion of standards that were previously developed and published by psychologist Dr. Rise VanFleet. The National Advisory Board included 14 members of the American Psychological Association Human-Animal Interactions Section 13 Division 17, with expertise from the fields of psychology, animal behavior, ethology, dog training, clinical social work, education, allied health, and occupational therapy.

DEFINITION OF ANIMAL ASSISTED INTERVENTIONS

(Reprinted with permission from Animal Assisted Interventions International)

Animal-Assisted Intervention

A goal-directed intervention is designed to promote improvement in physical, social, emotional and/or cognitive functioning of the person(s) involved and in which a specially trained animal handler team is an integral part.

AAI (Animal-Assisted Intervention) is directed and/or delivered by a practitioner with specialized expertise and within the scope of practice of his/her profession. There are specific goals for each individual involved and the process is documented and evaluated. The fields of Animal Assisted Education (AAE) and Animal-Assisted Therapy (AAT) cover these types of Animal Assisted Interventions. An intervention can also be less goal-directed, more casual or spontaneous. This type of intervention is designed to promote a range of other than therapeutic or educational benefits and to enhance the quality of life. The field of Animal-Assisted Activities (AAA) covers this type of Animal Assisted Interventions. An Animal-Assisted Intervention (AAI) may be provided in a variety of settings, may be group or individual in nature and may be implemented for persons of any age.

Animal-Assisted Therapy

An AAT intervention is formally goal-directed and designed to promote improvement in physical, social, emotional and/or cognitive functioning of the person(s) involved and in which a specially trained animal-¬handler team is an integral part of the treatment process. AAT (Animal-Assisted Therapy) is directed and/or delivered by a licensed/degreed (or equivalent) healthcare/human service professional with education and specialized expertise and within the scope of practice of his/her profession. AAT may be provided in a variety of settings, may be group or individual in nature and may be implemented for persons of any age. There are specific goals for each individual involved and the process is documented and evaluated.

Animal-Assisted Education

An AAE intervention is formally goal-directed and designed to promote improvement in cognitive functioning of the person(s) involved and in which a specially trained animal-handler team is an integral part of the educational process. AAE (Animal-Assisted Education) is directed and/or delivered by a licensed/degreed (or equivalent) professional with education, and specialized expertise within the scope of practice of his/her profession. AAE may be provided in a variety of settings, may be group or individual in nature and may be implemented for persons of any age. There are specific goals for each individual involved and the process is documented and evaluated.

Animal-Assisted Activity

An AAA intervention is less goal-directed as specific objectives may not be planned. AAA (Animal-Assisted Activity) is provided in a variety of settings, may be group or individual in nature and may be implemented for persons of any age. The AAA practitioners and/or animal handlers all are specially trained by an organization and meet the minimum standards set forth by AAII. Teams who provide AAA may also participate in Animal Assisted-Therapy (AAT) or Animal-Assisted Education (AAE) when the team is working directly with a healthcare, human service provider or with an educational practitioner.

Animal Support

AS (Animal Support) is not an intervention but is a support given by professional organizations that train animals and their handlers (examples: members of Assistance Dogs Europe, Assistance Dogs International, etc.). These organizations train animals to work alongside healthcare, social service, religious affiliations, or educational practitioners. In this way they participate in Animal Assisted Interventions.

ANIMAL WELFARE SPECIFIC TO AAI

Why are ethics for animals involved in AAIs different than other animal ethics? Existing language addressing animal welfare in psychology research is not generalizable to AAIs. In AAIs, animals are no longer our subjects. They work with us. They are our partners. Current perspectives of clinicians who utilize AAIs believe that animals should not be seen as "less than" or "tools" but as individuals with likes, dislikes, and limitations (Eccles, in press.) The rationale for this modification in perspectives is both practical and ethical because the welfare of the animal is inherently connected to the welfare of the client.

In the therapy setting, the clinician must be able to model empathy in their relationship with their animal, as this is an essential facet of a healthy relationship (VanFleet & Faa-Thompson, 2017). This relational power should represent a healthy model of leadership, support, and advocacy. Mutual understanding is contingent on the clinician's understanding of species-specific ethology and individual preferences of their animal partner.

By knowing and understanding species-specific ethology, individual behavior cues, body language, and the personality of the animals with whom they work, clinicians are better able to recognize and to intervene early when the animal becomes stressed or uncomfortable. Clinicians recognize that, like their clients, their animal partners might also be harmed by their participation in AAIs. It is the clinician that holds the responsibility of minimizing any negative impacts on the animal. The role of animals in clinical settings is inherently different from the role of animals in psychological research. Therefore, novel animal welfare guidelines and competencies are required.

HISTORY OF AAIs

The emergence of theories concerning the therapeutic benefits of animals for those with mental illness first emerged in the 18th century at the York Retreat, a mental institution in York, England. Inmates were permitted to wander the grounds freely, socializing with various small domestic animals (Serpell, 2006). The field of nursing also began to recognize the involvement of animals in treatment, when in the 1760s, Florence Nightingale, the founder of modern nursing, wrote about the benefits of integrating animals into patient care and recovery (Serpell, 2006). Another historical integration of animals in a therapeutic nursing setting occurred in 1944 in Pawling, New York. Patients who were recovering at the Army Air Corps Convalescent Hospital could work at the hospital farm, spurring future incorporation of animals in many therapeutic interventions (Jorgenson, 1997).

The 20th century also brought about the introduction of animals into the field of psychology. In the 1930s, Sigmund Freud integrated his Chow Chow dog Jofi, into sessions

with clients, primarily his adolescent clients. Freud reported the benefits of Jofi's interactions with clients where Jofi would lie close by, allowing the client to pet her (Coren, 2002, p. 139). In the early 1960s, child psychotherapist Boris Levinson discovered that one of his clients, a nonverbal 9-year-old, began to communicate when Levinson's dog attended psychotherapy sessions. He proceeded to incorporate his dog in sessions with other clients who had difficulty communicating and observed similar results (Coren, 2013). Around the same time as Levinson's practices with animals, Dr. Samuel Corson and his wife, Elizabeth, began promoting the inclusion of therapy dogs in a psychiatric facility and nursing home. Their research showed that dogs increased the social interactions of patients who were previously unresponsive to other treatments and improved patient-staff relations (Shubert, 2012).

In 1996, the Delta Society (now known as Pet Partners), one of the largest organizations responsible for the registration of therapy animals in the United States, published their "Standards of Practice for Animal-Assisted Therapy and Animal-Assisted Activities" in response to a lack of standardized guidelines concerning best practices for including animals in therapy. These standards addressed both the role of the handler (including continuing education, appropriate documentation of the animal's health, and demonstration of respect for and an ability to advocate for the animal) and requirements of the animal (including obedience and an ability to respond to general commands) (Fredrickson-MacNamara & Butler, 2006). Important to note in the evolution as well as the current practice of Animal-Assisted Interventions is the broad spectrum of respect for and inclusion of the animals in the process as partners versus tools for human use (Trevathan-Minnis & Shapiro, 2019, in press). The current document includes a list of guidelines, values, and professional approaches that respect the rights, comforts, and consent of animals in human-animal interactions and AAIs, and devalues those which incorporate animals as tools for human use.

Minimum criteria for practice:

Provider has demonstrated competency as a psychologist without an animal present as well as awareness that AAIs are an adjunct intervention rather than a stand-alone modality. APA General Principles: At a foundational level, the HAI Division competencies build upon the General Principles of APA that include Principle A: Beneficence and Nonmaleficence, Principle B: Fidelity and Responsibility, Principle C: Integrity, Principle D: Justice and Principle E: Respect for People's Rights and Dignity and rely on the provider to ensure these principles are followed.

APA Ethical Standard 2.01 Boundaries of Competence:

APA Ethical Standard 2.01 Boundaries of Competence will provide the general framework from which Human-Animal Interventions competencies are developed. Essentially, Boundaries of Competence rely on the practitioner to serve, teach and conduct research only within areas and with populations where they have been educated, trained, supervised or have experienced and are able to apply cultural sensitivity. When scientific or professional knowledge is limited, reasonable efforts must be made to ensure competence of practice in order to protect clients/patients and those under their charge from harm; and becoming familiar with administrative or judicial rules. As with other benchmarks, these standards are meant to provide a resource and guidance in the practice of Human-Animal Interventions.

STANDARDS (APA Standard 2.01: Competence):

As previously noted, this section builds upon APA Standard 2.01 Competence as well as previous research in the area of AAI competencies as published by Stewart et al., 2016. Adding a sentient being to clinical practices requires broader Knowledge, Skills and Attitudes (KSAs) than traditionally educated professionals. This document shares the minimum KSAs recommended to practice as a competent provider of AAIs using the six APA 2.01 clusters with competencies for each area. The KSAs listed are meant to supplement

existing standards by specifying constructs unique to AAIs. In addition to these competencies, providers are encouraged to be familiar with guidelines published by other international organizations, such as the International Association for Human-Animal Interaction Organizations (IAHAIO) and Animal-Assisted Intervention International (AAII).

WHAT ARE KSAs

Within this document, Knowledge, Skills, and Attitudes refer to the gaining of relevant, reputable information (Knowledge), the application of that knowledge in a practical setting (Skills) and a valuing of the need for related supervision, advocacy and continued professional development (Attitudes). The APA Standards clusters are listed below (Table S1).

Table S1. APA Standards Clusters

(1)	Professional	Values and attitudes Cultural considerations
(2)	Relational	Interdisciplinary Teams
(3)	Science	Related to Animal Assisted Interventions and the Human Animal Bond
(4)	Application	Interventions<br>Consultation
(5)	Education	Teaching<br>Supervision
(6)	Systems	Leadership<br>Consultation<br>Interprofessional team

I. Professionalism (Professional Values/Attitudes)

Professionalism as a psychologist has already been established upon graduation. Adding the adjunctive modality of Animal-Assisted Interventions means not only having a professional identity as a psychologist, but also as a practitioner who values professionalism as an AAI provider through Integrity, Deportment (conduct), Accountability, Concern for the Welfare of Others, Cultural Sensitivity, Ethical Attitudes, Reflective Practice, Displaying Core

Competencies, and participation in a supervisory process specific to AAIs.

Knowledge, Skills and Attitudes in Professionalism Professional Competencies are evidenced by, but not limited to:

(A) Ethical codes
- (a) Relevant ethical and professional codes and guidelines for the safety of animals and humans in sessions from reputable sources in AAIs
- (b) Clinicians acting as role models of healthy, appropriate, safe interactions with both animals and humans
- (c) Awareness of and ability to negotiate the multiple relationships that are inherent in AAIs (client/therapy animal, therapy animal/clinician, client/clinician)
- (d) Self-reflection, peer consultation and supervision
- (e) Giving animals a choice at all times to opt out of participation

(B) Personal and cultural biases
- (a) Personal and professional factors and motivations including transference, countertransference, and personal bias
- (b) Influences that may impact the decision to incorporate AAIs (including the animal's suitability)
- (c) Biases about therapy animals and one's bond with the animal

(C) Risk management
- (a) Risk management strategies and skills
- (b) General, personal and professional liability
- (c) Legal issues
- (d) Appropriate documentation (informed consent, treatment plans, progress notes)

(e) Zoonotic procedures

(D) Education
- (a) AAI-related education and training
- (b) Continuing education/professional development
- (c) Current literature and evidence-based practices

(E) Animal Behavior
- (a) Species, breed, and individual behaviors
- (b) Respect for each animal's age, health, physiology, temperament, ability, and stamina
- (c) Evaluate each animal's suitability on a continuous, on-going basis
- (d) Identify a retirement process for the therapy animal; provide a closure process for clients

II. Relational

Relational competencies are determined by how effectively psychologists interact with their clients, patients, students, and research participants as well as with their therapy animal(s). Adding a sentient being to the therapeutic session creates a triangular relationship (referred to as the Treatment Triad) rather than a bi-directional one. Having an additional partner, particularly one who does not speak the same language, requires adept skills of the psychologist in order to split attention, maintain desired outcomes, and provide a strong, here-and-now presence throughout the session. These Relational Competencies are evidenced by displaying interpersonal skills and expressive skills when communicating ideas and feelings in both verbal and nonverbal ways. Further, Relational Competencies may help manage conflict.

Knowledge, Skills and Attitudes in Relational Competencies Relational competencies are evidenced by, but not limited to:

(A) Animal Behavior
- (a) Signs of positive emotional, physiological, and physical well-being
- (b) Signs of stress
- (c) Ways to ameliorate stress
- (d) Education and training related to species-specific knowledge and skills

(B) Animal Training
- (a) Extends 'do no harm' tenet to training and interaction methods with therapy animals
- (b) Follows the Five Freedoms of Animal Welfare (at a minimum) as well as Mellor's Quality of Life Scale (2016) for optimum animal
- (c) welfare
- (d) Uses positive training methods to foster learning without pain, fear, or coercion, and subsequently harming the relationship

(C) AAI Techniques
- (a) Includes a theoretical framework that ensures intentionality in practice
- (b) Knowledge of a variety of theoretical orientations and applies them to AAI
- (c) Continuously monitors for signs of stress, therapeutic moments

(D) Animal Welfare
- (a) Provides intentional time away from AAI as well as limits the amount of time at work according to animal stamina and well-being
- (b) Allows time and space for animal's reprieve
- (c) Therapy animals are able to maintain species typical behaviors, such as having access to water available at all times or grazing behaviors if desired
- (d) Recognizes that interactions that are not mutually beneficial could be considered animal exploitation and avoids such interactions
- (e) Allows the animal a constant consent process where the animal gets to choose when and with whom to interact
- (f) Continuously assesses for signs of willingness to engage in the therapeutic intervention
- (g) Continuously assesses for physical and medical limitations in the animal and intervene as quickly as possible

III. Science

Competencies related to Science are specific to AAI research methodology, outcomes, and humane techniques in data collection and analysis. Research topics related to AAIs

include human and animal biology, behavior, psychology, cognitive-affective perspectives and an overall respect for science and AAIs. The science does not necessarily apply to the daily interactions between client, practitioner, and animal, but does include the application of evidence-based practices. Additionally, if the psychologist does pursue research-related endeavors, the animal behavior and animal welfare sides are not minimized or dismissed. Items related to training, behavior, and zoonoses have been addressed above in the Relational section which may overlap in this section and should not be considered to occur in isolation.

These Science Competencies are evidenced by displaying critical thinking, understanding the scientific foundation of practice, and applying scientific methods to practice. These guidelines are specific to studies in human-animal interactions and are separate from animal research in other contexts such as medical research.

Knowledge, Skills and Attitudes in Science Science competencies are evidenced by, but not limited to:

(A) Animal Behavior and Welfare
- (a) Uses behavioral ethology
- (b) Understands the science of animal training, including but not limited to operant and classical conditioning
- (c) Uses positive and humane training principles consistent with best practices

(B) Non-Research
- (a) Follows IRB processes to ensure the safety and well-being of animals
- (b) Follows IRB processes to ensure the safety and well-being of participants
- (c) Follows all relevant laws and guidelines (e.g., Institutional Animal Care and Use Committee (IACUC) at the international, federal, state, and local levels)
- (d) Has familiarity with current research related to AAIs

(C) Terminology
- (a) Recommends using standard consistent AAI terminology from organizations that are aligned with these APA competencies (e.g International Association of Human Animal Intervention Organization, Animal Assisted Intervention International, Professional Association Therapeutic Horsemanship (PATH), and Pet Partners

IV. Application

Application is the integration of evidence-based practices. While the Science (III) section focuses on the knowledge of empirical evidence, Application (IV) refers to the conduct of such practices. In addition to assessments, it includes clinical decision-making, writing progress notes, taking into account behaviors of the therapy animals and communicating these to the client, intervention planning, conceptualizing cases, and planning sessions using at least one consistent theoretical orientation. Additionally, the AAI clinician is required to keep appropriate documentation that includes an informed consent and AAI-specific notes that include animal reactions, behaviors both within and outside of sessions, and the animal's functional abilities.

These Applicational Competencies are evidenced by inclusion of best practices in scientific, theoretical, and contextual assessment and interventions as well as the assessment and diagnosis of presenting symptoms. On the human side, most of this is done through the practitioner's professional identity. In some cases, evaluating a client for suitability is required and the practitioner has further education, training, and experience specific to AAIs. The AAI clinician develops session plans with intentionality and determines client progress.

Knowledge, Skills and Attitudes in Application

(A) Therapy Animal Training
- (a) Understands and implements humane animal training tools, methods, and equipment
- (b) Uses accurate behavioral terminology and positive reinforcement animal training methods

(B) Handler Disposition and Training
- (a) Has applicable knowledge and training in animal behavior (species, breed and individual levels)

(b) Complies with national therapy animal evaluation and registration guidelines
(c) Exhibits a calm demeanor during interventions, recognizing that the handler's affect impacts the animal, client and interaction
(d) Demonstrates proficiency in the use of appropriate animal-related equipment (harnesses, leashes, ropes, etc.)
(e) Demonstrates proficiency in cue delivery and expectation to therapy animals
(f) Remediates signs of stress in the therapy animal promptly
(g) Understands the power-differential within the relationship between the therapy animal and clinician
(h) Objectively protects the animal's optimal welfare in spite of the client's or clinician's preferences.
(i) Knowledge of zoonotic diseases and prevention of these transmittable diseases

(C) Documentation and Informed Consent
(a) Develops and implements AAI-specific informed consent forms
(b) Develops and implements AAI-specific treatment plans and session planning using measurable and objective interventions in treatment goals
(c) Maintains progress notes that include behaviors of the therapy animal and the interactions between patient and therapy animal while referencing a specific AAI

(D) Assessment/Evaluation
(a) Uses a screening protocol to determine if AAI is appropriate for both the patient and the therapy animal
(b) Recognizes therapy animal signs of readiness, consent and non-consent, and responds by respecting the animal's choice
(c) Recognizes the client's signs of readiness, consent and non-consent to the inclusion of a therapy animal
(d) Understands why the animal is in the session, as reflected in the clinician's intentionality in practice
(e) Discusses purpose and implications to clients and/or parents or guardians (as needed)
(f) Reassesses treatment plan for appropriateness on a regular basis

(E) Techniques
(a) Knowledge and application of how AAIs are in alignment with chosen theoretical orientation(s)
(b) Applies specific AAI techniques and principles
(c) Understands implications of AAI for specific client populations (outcomes, goal setting)
(d) Practices flexibility, openness, and creativity in sessions
(e) Responds quickly and appropriately to unexpected animal behavior as well as risky or intrusive client-animal behavior
(f) Understands risks associated with the inclusion of a therapy animal in practice from safety, legal, ethical and cultural competency levels

(F) Theories
(a) Uses consistent theoretical orientations in AAI sessions
(b) Knowledge and application of AAI specific theories

V. Education/Training

Education is providing instruction about AAIs to others that includes both the dissemination of knowledge and evaluating the knowledge and skills to future AAI providers through the teaching of courses and workshops. The Education/Training Competencies are evidenced by knowledge of teaching methods, the application of teaching methods in a variety of settings, supervision. and monitoring the professional functioning of others. Furthermore, the Application Cluster refers to using the latest evidence-based interventions in AAIs as well as instruction of others. Educators and trainers should already meet all of the other competencies and guidelines themselves before teaching others.

Knowledge, Skills and Attitudes in Education/Training Education and training competencies are evidenced by, but not limited to:

(A) Literature
(a) Understands the history of AAIs and the human-animal bond across the lifespan
(b) Knows where to find scholarly readings regarding efficacy and outcomes in AAIs
(c) Analyzes the strength of available evidence
(d) Adheres to IRB guidelines for humans and animals

(e) Acknowledges own limitations and clinical expertise before deviating from evidence-based or best practices

(B) Values
- (a) Values and commits to lifelong learning about both animals and clients
- (b) Empowers and supports learning opportunities for students and trainees
- (c) Speaks to and educates groups, institutions, organizations within one's skillset and scope of practice
- (d) Collaborates or consults with other AAI professionals

VI. Systems

The System Cluster refers to a system in which members of varying disciplines interact with and confer on issues relative to meeting the therapeutic goals of an individual being served across those disciplines. In the Systems cluster, professional identity as a psychologist has already been achieved based on the APA Code of Ethics and licensure. The interdisciplinary system refers to knowledge related to issues about a variety of disciplines and the ability to interact with multiple disciplines. Along with the treatment triad and various disciplines involved, the animal is considered a stakeholder in the system. Administration manages the delivery of services or administration of organizations, agencies or programs, consultation with organizations, and advocacy. Advocacy provides an additional layer of advocacy to any animals incorporated into those services. The psychologist is already required to advocate for clients, adding AAIs means advocating for the therapy animal as well. Advocacy for the animal occurs continuously because the animal has the least power in the system. A stressed or scared animal might negatively impact the session and outcomes, as well as tarnish the relationship between the handler/owner/client/therapy animal, the needs of the animal takes precedence for the safety of everyone.

Knowledge, Skills and Attitudes in Systems.

Systems competencies are evidenced by, but not limited to:

(A) Resources
- (a) Is familiar with resources for ongoing education about animal species participants
- (b) Is familiar with the system roles and responsibilities

(B) Collaboration with and respect for other professions
- (a) Interdisciplinary teams in treatment (including animal-related fields) in treatment planning and goal setting
- (b) Basic knowledge of roles, responsibilities, and objectives for a variety of different professionals, including animal related professions
- (c) Consideration and inclusion of theories, ideas and input from the team

(C) Consultation with organizations
- (a) Playing a role regarding establishing a practice
- (b) Familiarity with how AAIs fit within a system
- (c) Consultation is conducted within the scope of practice

(D) Animal welfare
- (a) AAI documentation (case notes) is completed by every member of the team

References

1. Allen, K.; Colbert, L. Ethical and safety considerations for use of animals in a therapeutic setting. *Psychother. Bull.* **2016**, *51*, 35–45.
2. Beck, A.M.; Katcher, A.H. Future directions in human-animal bond research. *Am. Behav. Sci.* **2003**, *47*, 79–93.
3. Coren, S. *The Pawprints of History: Dogs in the Course of Human Events*; Free Press: New York, NY, USA, 2002.
4. Coren, S. How Therapy Dogs Almost Never Came to Exist. Psychology Today. 11 February 2013. Available online: psychologytoday.com. (accessed on 30 September 2018).
5. Eccles, E.P. Ethical guidelines for working with animals in clinical settings. *ProQuest Dissertations & Theses Global*. 2020, 1–163. Available online: https://search.proquest.com/docview/2386182145 (accessed on November 10, 2021).
6. Evans, N.; Gray, C. The practice and ethics of animal-assisted therapy with children and young people: Is it enough that we don't eat our co-workers? *Br. J. Soc. Work* **2012**, *42*, 600–617.
7. Fine, A.H.; (Ed.) Handbook on Animal-Assisted Therapy: Foundations and Guidelines for Animal-Assisted Interventions; Academic Press: Cambridge, MA, USA, 2019.

8. Fredrickson-MacNamara, M.; Butler, K. The art of animal selection for animal-assisted activity and therapy programs. In *Handbook on Animal-Assisted Therapy: Theoretical Foundations and Guidelines for Practice*, 2nd ed.; Fine, A.H., Ed.; Academic Press: Amsterdam, The Netherlands, 2006; pp. 3–20.
9. Jorgenson, J. Therapeutic use of companion animals in health care. *J. Nurs. Scholarsh.* **1997**, *29*, 249–254.
10. Mellor, D. Updating animal welfare thinking: Moving beyond the "Five Freedoms" towards "a Life Worth Living". *Animals* **2016**, *6*, 21. https://doi.org/10.3390/ani6030021.
11. Ng, Z. Advocacy and rethinking our relationships with animals: Ethical responsibilities and competencies in animal-assisted interventions. In *Transforming Trauma: Resilience and healing through our connections with animals*; Purdue University, West Lafayette, IN, USA, 2019; pp 55-90.
12. Serpell, J.A. Animal-assisted interventions in historical perspective. . In *Handbook on Animal-Assisted Therapy: Theoretical Foundations and Guidelines for Practice*, 2nd ed.; Fine, A.H., Ed.; Academic Press: Amsterdam, The Netherlands, 2006.
13. Shubert, J. Dogs and human health/mental health: From the pleasure of their company to the benefits of their assistance. *U.S. Army Med Dep. J.* **2012**, (2),21–29.
14. Stewart, L.A.; Change, C.Y.; Parker, L.K.; Grubbs, N. *Animal-Assisted Therapy in Counseling Competencies*; American Counseling Association, Animal-Assisted Therapy in Mental Health Interest Network: Alexandria, VA, USA, 2016
15. Tedeschi, P.; Fitchett, J.; Molidor, C.E. The incorporation of animal-assisted intervention in social work education. *J. Fam. Soc. Work* **2005**, *94*, 59–77.
16. Trevathan-Minnis, M.; Shapiro, K. Human-animal studies in psychology: The history and challenges of developing clinically-based, ethical programs involving animals. *Humanist. Psychologist, online first*. **2021**.
17. VanFleet, R.; Faa-Thompson, T. *Animal Assisted Play Therapy*; Professional Resource Press: Sarasota, FL, USA, 2017.
